# Supplementary material for: Control of spin-wave transmission by a programmable domain wall
Source: Nat Commun. 2018 Nov 19;9:4853. doi: 10.1038/s41467-018-07372-x (PMC6242868; doi:10.1038/s41467-018-07372-x)
Supplement: Supplementary file 1 — Supplementary Information [file 41467_2018_7372_MOESM1_ESM.pdf]

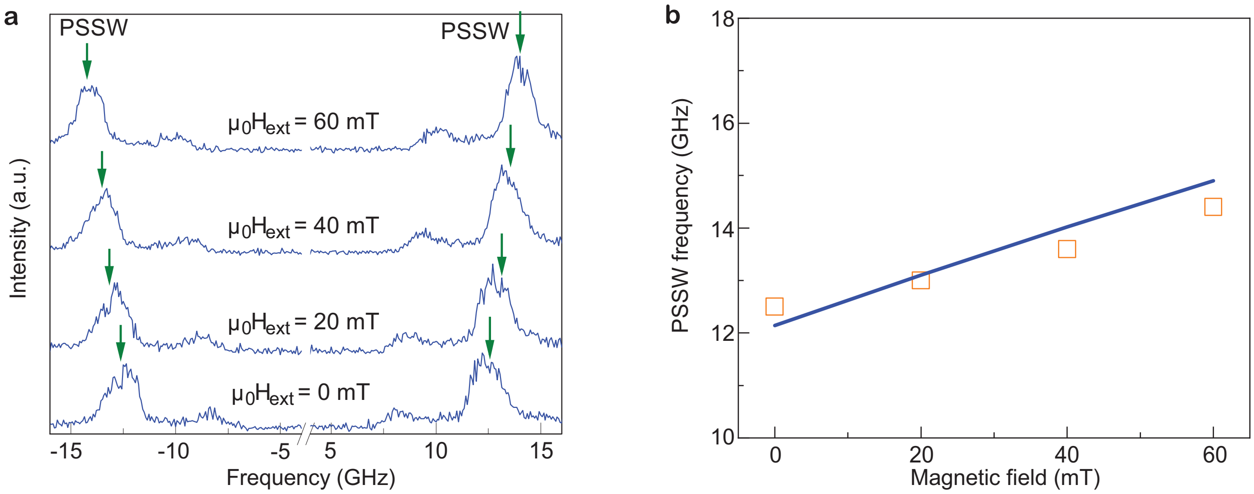

Supplementary Figure 1. **a** μ-BLS spectra recorded at a fixed position of the CoFeB/$BaTiO_3$ sample and with the microwave antenna turned off. The spectra are measured with different external magnetic fields, as indicated in the graph. **b** Comparison between the measured (squares) and calculated (blue line) values of the PSSW frequency as a function of applied magnetic field. For the calculation we used $f_{\rm PSSW} = \frac{\gamma\mu_0}{2\pi}\sqrt{\left[H_{\rm ext}+H_{\rm ani}+\frac{2A_{\rm ex}}{M_{\rm s}}\left(\frac{p\pi}{d}\right)^2\right]\left[H_{\rm ext}+H_{\rm ani}+\frac{2A_{\rm ex}}{M_{\rm s}}\left(\frac{p\pi}{d}\right)^2+M_{\rm s}\right]}$ and input parameters; exchange constant $A_{\rm ex} = 1.35\times10^{-11}$ J/m, saturation magnetization $M_{\rm s} = 1.15\times10^{6}$ A/m, uniaxial magnetic anisotropy $K_{\rm u} = 2.5\times10^{4}$ J/m^3, CoFeB film thickness d = 50 nm, and $H_{\rm ani} = 2K_{\rm u}/M_{\rm s}$. From the data in (**a**) and (**b**) we conclude that a thermally-excited first-order PSSW mode produces the peaks at ±12.6 GHz in Fig. 2b of the main manuscript.

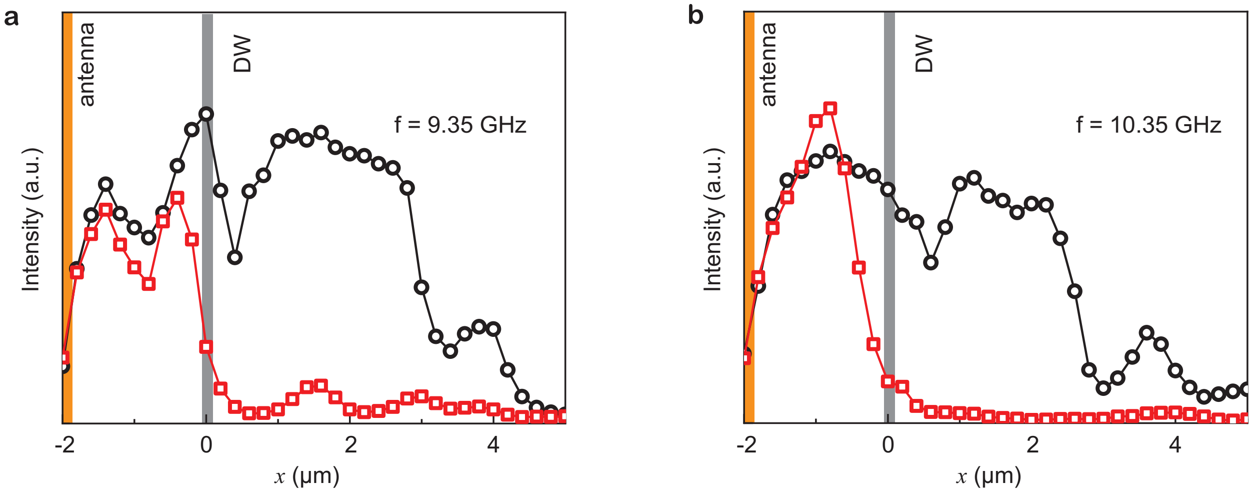

Supplementary Figure 2. Line scans of BLS intensity recorded from the antenna edge across the pinned domain wall at an excitation frequency of 9.35 GHz (**a**) and 10.35 GHz (**b**). The black and red curves indicate data for a broad 90° head-to-head and narrow 90° head-to-tail domain wall, respectively.

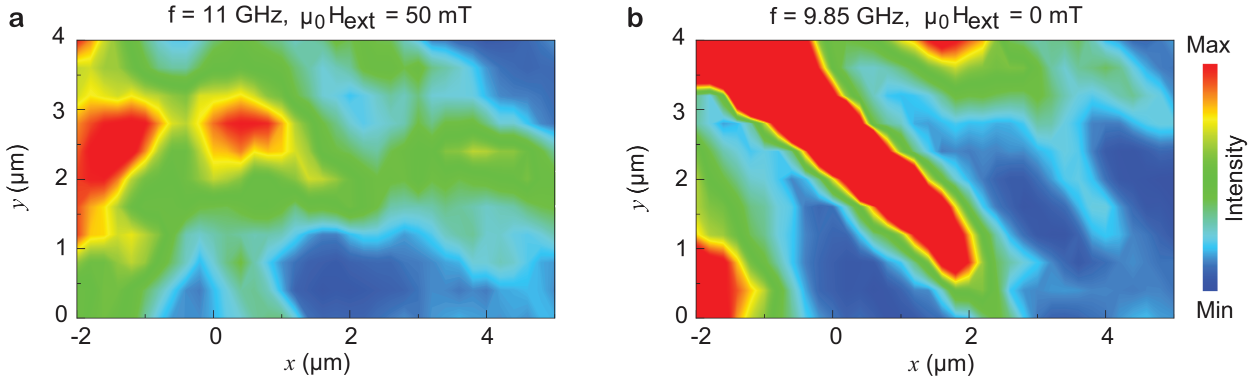

Supplementary Figure 3. Areal maps of BLS intensity recorded in a magnetic field of 50 mT (**a**) and in zero field on an area of the sample without domain wall (**b**). The field in (**a**) saturates the magnetization, i.e., it is large enough to erase the domain wall at $x = 0$ μm.

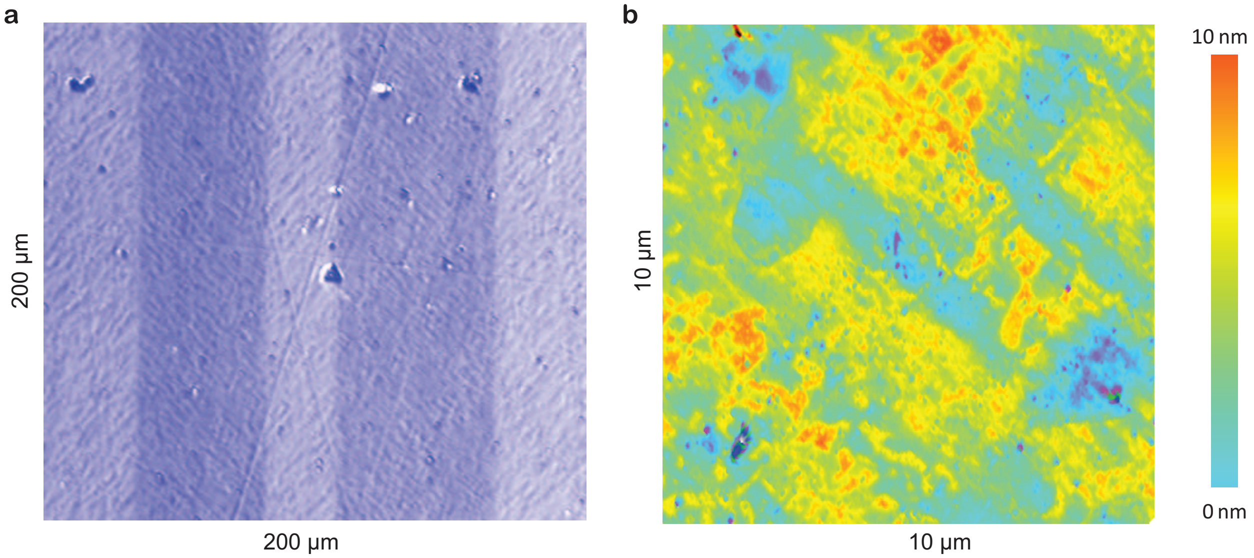

Supplementary Figure 4. Optical microscopy image (**a**) and atomic-force microscopy image (**b**) of a 50-nm-thick CoFeB film on $BaTiO_3$. The images demonstrate the granular structure of the CoFeB film.
