# Supplementary material for: Control of spin-wave transmission by a programmable domain wall
Source: Nat Commun. 2018 Nov 19;9:4853. doi: 10.1038/s41467-018-07372-x (PMC6242868; doi:10.1038/s41467-018-07372-x)
Supplement: Supplementary file 3 — Description of Additional Supplementary Files [file 41467_2018_7372_MOESM3_ESM.pdf]

Description of Additional Supplementary Files

File Name: Supplementary Movie 1
Description: Spin-wave reflection from a resonance mode in a narrow head-to-tail domain wall. The resonance mode is characterized by two oscillatory out-of-phase antinodes on opposite sides of the domain wall center.
