# Supplementary figures and images for: Control of spin-wave transmission by a programmable domain wall
Source: Nat Commun. 2018 Nov 19;9:4853. doi: 10.1038/s41467-018-07372-x (PMC6242868; doi:10.1038/s41467-018-07372-x)

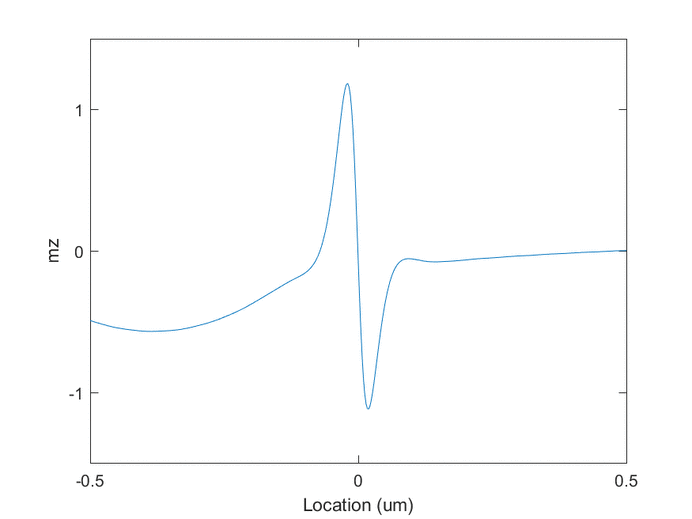

Supplement: Supplementary file 4 — Supplementary Movie 1 [file 41467_2018_7372_MOESM4_ESM.gif]
